# Draft Genome Sequence of the Antibiotic-Producing Cystic Fibrosis Isolate *Pantoea agglomerans* Tx10

**DOI:** 10.1128/genomeA.00904-13

**Published:** 2013-10-31

**Authors:** Derek D. N. Smith, Morgan W. B. Kirzinger, John Stavrinides

**Affiliations:** Department of Biology, University of Regina, Regina, Saskatchewan, Canada

## Abstract

*Pantoea agglomerans* is an enteric bacterium that is capable of causing both plant and human disease. Here, we report the genome sequence of a cystic fibrosis isolate, *P. agglomerans* Tx10, which produces an antibiotic that is effective against *Staphylococcus aureus*.

## GENOME ANNOUNCEMENT

*Pantoea agglomerans* (formerly *Enterobacter agglomerans* and *Erwinia herbicola*) (1) is a member of the *Enterobacteriaceae* that has been noted to cause plant disease, as well as opportunistic infections in humans (2–4). *P. agglomerans* has also been shown to have unique metabolic capabilities, including antibiotic biosynthesis (4–10). Among these antibiotics are pantocins (11–14), herbicolins (15, 16), microcins (17–19), and phenazines (20), several of which target amino acid biosynthesis in the fire blight pathogen, *Erwinia amylovora*, and have been developed into biocontrol agents. Here, we report the complete genome sequence of the clinical isolate *P. agglomerans* Tx10, which was isolated from the sputum of a cystic fibrosis patient. This isolate produces multiple antibiotics that target *E. amylovora* and clinically relevant pathogens, including *Staphylococcus aureus, Streptococcus epidermidis*, and *Escherichia coli*.

Total DNA was sequenced using Illumina HiSeq 2000, 100-bp paired-end sequencing, resulting in 17,035,538 reads, with an average Phred quality score of 31. ABySS version 1.3.5 (21) was used for *de novo* paired-end assembly using the default parameters and an optimized *k*-mer value of 83. This resulted in 38 contigs with an N_50_ of 586,961 bp and an estimated genome size of 4,856,603 bp at 347× coverage. Contigs of ≥200 bp (25 total) were submitted to the NCBI Prokaryotic Genome Automatic Annotation Pipeline version 2.0, resulting in 4,627 predicted genes. Of these, there are 4,500 predicted coding genes, 32 pseudogenes, 24 rRNAs, and 71 tRNAs. Two contigs are predicted to represent plasmids of 173,724 bp and 681,148 bp.

The *P. agglomerans* Tx10 genome provides the means not only for identifying antibiotic biosynthetic clusters but also for evaluating the contribution of natural products to polymicrobial infections in cystic fibrosis.

### Nucleotide sequence accession numbers.

This whole-genome shotgun project has been deposited at GenBank under the accession no. ASJI00000000. The version described in this paper is the first version, ASJI01000000.
